# Adjustment to COVID-19 Lockdown Among Italian University Students: The Role of Concerns, Change in Peer and Family Relationships and in Learning Skills, Emotional, and Academic Self-Efficacy on Depressive Symptoms

**DOI:** 10.3389/fpsyg.2021.643088

**Published:** 2021-08-19

**Authors:** Emanuela Calandri, Federica Graziano, Tatiana Begotti, Elena Cattelino, Silvia Gattino, Chiara Rollero, Angela Fedi

**Affiliations:** ^1^Department of Psychology, University of Torino, Torino, Italy; ^2^Department of Human and Social Sciences, University of Aosta Valley, Aosta, Italy

**Keywords:** COVID-19 lockdown, young adults, depressive symptoms, family relationships, peer relationships, emotional self-efficacy, academic self-efficacy

## Abstract

In Italy strict containment measures against COVID-19 pandemic were implemented from March to May 2020 with home confinement and schools and universities closed. Students shifted to remote learning, experienced a forced isolation from peers and an increased sharing of time and spaces with the family. The influence of these aspects on the psychological adjustment of university students is largely unexplored. This paper was aimed at investigating the role of some correlates of depressive symptoms specific to the lockdown condition experienced by young university students, namely contagion concern, perceived worsening of family, and peer relationships and perceived worsening of learning skills. Moreover, the possible mediating effects of emotional and academic self-efficacy in these relationships were examined. Data were collected from 296 Italian university students (aged 18–25 years; 83% female students) through an online survey by means of a snowball sampling methodology in May 2020. Significant depressive symptoms were reported by 67% of participants. Contagion concerns were related to depressive symptoms through the mediating effect of emotional self-efficacy. Worsening of learning skills was related to depressive symptoms through the mediating effect of academic self-efficacy. Depressive symptoms were directly related to worsening of family relationships but unrelated to worsening of peer relationships. Results are discussed in relation to the need of preventive interventions for this specific population in view of academic activities planning in the post-COVID 19 period.

## Introduction

In early 2020 Italy was the first western country to experience the COVID-19 pandemic outbreak. Since 9 March 2020, all schools and universities have been closed and remote learning has been activated, and at present university students still do not have face-to-face classes (except for a short period in September and October 2020). In particular, from 9 March to 3 May 2020, Italy lived a strict lockdown status, all activities were closed except essential services. For adolescents and young adults this confinement caused several changes in their daily lives. The closure of schools and universities and the interruption of leisure activities have affected habitual routines, forcing young people to share a lot of time with their families and, on the contrary, interrupting face-to-face relationships with peers. This is a condition never experienced before by most of the current Western population, whose psychological effects are now being studied all over the world. In recent months, the number of publications on the effects of this pandemic has risen sharply, but there are still few publications focused on young adults.

Referring to the life-span developmental perspective (Baltes, 1997; Elder and Shanahan, 2006) and focusing on young adulthood as theorized by Arnett (2000), our study aimed to investigate some aspects of the psychological adjustment of Italian university students to COVID-19 and containment measures through a cross-sectional research carried out in May 2020, during the last month of lockdown.

Lockdown conditions have represented a combination of changes that can make this population particularly vulnerable, as some studies indicate. Several studies found a significant increase in psychological problems (Brooks et al., 2020; Liang et al., 2020), in particular depressive symptoms (Meda et al., 2021), stress, irritability, anger (Shanahan et al., 2020; Taylor et al., 2020), anxiety related to the concerns of contagion (Mazza et al., 2020; Schiff et al., 2020; Xiang et al., 2020). Moreover, these psychological difficulties, in particular depressive symptoms, were found to be more common among young adult women than men (Ettman et al., 2020). Literature reported a female preponderance of depressive symptoms during young adulthood in normative situations (Beirão et al., 2020), thus this gender difference seems to hold true also during the non-normative situation of the pandemic. This paper was aimed at analyzing the role of some correlates of depressive symptoms specific to the lockdown condition experienced by young university students, namely contagion concern, perceived worsening of family and peer relationships and perceived worsening of learning skills. Moreover, the possible mediating effects of emotional and academic self-efficacy in these relationships were examined.

As stressed by Schiff et al. (2020) the COVID-19 related concerns represented a unique combination of worries related to both objective fears and subjective anxiety. University students' concerns about personal and parents' health represented one of the potential correlates of difficulties in functioning. In particular, the new perceived responsibility of taking care and protecting their relatives from being infected could have a significant impact on students' mental health.

Not only the contagion concern, but also the consequences of isolation can promote depressive symptoms. The imposed home confinement can have a significant effect especially for young adults (18–25 years old) who are experiencing a period of the life span characterized by specific developmental tasks, in particular a strong need of independence from parents, a redefinition of relationships with peers, and a need of making choices about future studies and job career (Santrock, 2019). The unprecedented situation of the lockdown represented a non-normative event and a developmental challenge in the life span (Hendry and Kloep, 2002), in which young adults were likely to experience difficulties in family, peer, and academic contexts (Fioretti et al., 2020).

Although the pivotal role of the family during youth has been extensively documented in the literature, the continuous cohabitation imposed by the lockdown may play a critical role. As stressed by Collins and Laursen (2004), during times of crisis parents can both intensify or mitigate the impact of stressful experiences on young people's mental health. In particular, during the imposed home confinement parents could offer support and contribute to create adaptive experiences or, on the contrary, their own concern about the pandemic and its effects could negatively affect youth adjustment (Ellis et al., 2020). Moreover, the interruption of physical relationships with peers may have significant consequences in terms of social status, sense of belonging to peer-group and lack of social and emotional support (Ellis et al., 2020; Elmer et al., 2020). Regarding academic activities during the lockdown, remote learning represented a new situation and many students experienced it as a major challenge, as they were largely unprepared (Blume et al., 2020). The online learning required new ways of studying and organizing one's own didactic practice to deal efficiently with academic tasks. Developmental psychology studies highlighted that problems in family relationships, social isolation from peers, and academic difficulties are all risk factors for depressive symptoms in youth (Kassis et al., 2017; Shore et al., 2018; Calandri et al., 2019; Schwartz-Mette et al., 2020).

As stated before, we were interested in examining the role of contagion concern, perceived worsening of family and peer relationships, and perceived worsening of learning skills on depressive symptoms with a view to a possible mediating effect of self-efficacy. Perceived self-efficacy consists in the personal belief to be able to face new situations, difficulties, and challenges (Bandura, 1997). In particular, emotional self-efficacy refers to the perceived abilities to regulate negative and positive emotions (Bandura et al., 2003), whereas academic self-efficacy refers to the perceived abilities to manage challenges and difficulties related with studying activities (Bassi et al., 2007). Self-efficacy is influenced by mastery experiences and emotions and self-efficacy in turn affects emotional experiences (Bandura, 1997; Usher and Pajares, 2008). In particular, both emotional and academic self-efficacy proved to have a pivotal role in managing stressors and protecting young people from depressive symptoms (Bandura et al., 2003; Calandri et al., 2021). We chose to examine the role of emotional and academic self-efficacy because during the lockdown, young students had to manage negative emotions related to the fear of contagion and to home confinement. Moreover, they had to face remote learning, a completely new situation for which they had no previous experience of effective management (Besser et al., 2020). In particular, remote learning required new ways of regulating learning activities in order to face academic demands. These aspects are expected to influence students' self-efficacy beliefs and these in turn might be related to depressive symptoms.

### Aims and Hypotheses

The present study examined the role of some correlates of depressive symptoms of young university students during the first COVID-19 Italian lockdown (March–May 2020), namely contagion concern, perceived worsening of family and peer relationships, and perceived worsening of learning skills. Moreover, emotional and academic self-efficacy were assumed as mediating variables in the relationships between these correlates and depressive symptoms.

The aims of this study were:

1. To describe COVID-19 contagion concern, perceived changes in family and peer relationships and in learning skills, levels of emotional, and academic self-efficacy and the presence of depressive symptoms in a group of university students, examining gender differences. This analysis was exploratory, and no specific assumptions were made, except for depressive symptoms which were expected to be higher among young adult women compared to men in the light of the literature reviewed above (Beirão et al., 2020; Ettman et al., 2020).
2. To investigate the role of COVID-19 contagion concern, worsening of family and peer relationships and worsening of learning skills on depressive symptoms and to explore the possible mediating role of emotional and academic self-efficacy.
  In particular, we hypothesized a mediating role of emotional self-efficacy between:
  a) COVID-19 contagion concern and depressive symptoms;
  b) Worsening of family relationships and depressive symptoms;
  c) Worsening of peer relationships and depressive symptoms;
Moreover, we hypothesized a mediating role of both emotional and academic self-efficacy between:
  d) Worsening of learning skills and depressive symptoms.

The mediation models were tested including gender as a covariate to control its effect on depressive symptoms.

## Materials and Methods

### Participants and Procedure

The present study was implemented by two Italian universities: University of Torino and University of Aosta Valley. Data were gathered through an online questionnaire (on the Limesurvey platform) by means of a convenience sampling technique (snowball sampling): each research team recruited participants sending the survey link to university students' mailing lists and to direct contacts in their geographical area. Respondents in turn were invited to share the link with their contacts. Inclusion criteria were being a university student and living in Italy. Students provided online informed consent and confirmed their voluntary participation to the study. Respondents did not receive any incentive for their participation. Data were collected in May 2020, during the last month of lockdown. The study was approved by the Bioethics Committee of the University of Torino.

After deleting 44 incomplete responses, a total of 296 valid questionnaires were analyzed in this study. Students' age ranged from 18 to 25 years old (mean age = 20.9 years, *sd* = 1.3) and most participants were female students (83%). Most students lived with parents (92%), in line with Italian data (EUROSTAT, 2019). Most participants came from Northern Italy (94%), the geographical area most affected by the pandemic, although the whole national territory was in lockdown when data were gathered. Most participants were psychology students (45.6%) attending the first year of university (37.6%). The mean daily amount of time spent on remote learning was 5 h (*sd* = 2) (Table 1).

**Table 1:** Characteristics of study participants (*N* = 296).

	***N***	**%**
Gender		
Male	50	17
Female	246	83
Age, *mean* (*sd*)	20.9 (1.3)	
Course of study		
Psychology	135	45.6
Humanities/economics/law	77	26.0
Health and social sciences	41	13.9
Sciences/math/engineering	31	10.5
Missing	12	4.0
Academic year		
First	111	37.6
Second	96	32.4
Third	83	28.0
Fourth	5	1.7
Fifth	1	0.3
Remote learning (hours per day), *mean* (*sd*)	5.0 (2.0)	
Devices for remote learning^a^		
Personal computer	259	87.5
Smartphone	66	22.3
Tablet	54	18
Living situation		
With both parents	211	72
With one parent	59	20.1
Other (alone/with peers/with partner)	23	7.8
Missing	3	0.1

a *The total is higher than 100% because more answers were possible*.

### Measures

The questionnaire included validated measures to evaluate depressive symptoms, emotional, and academic self-efficacy, as well as specific questions specifically developed for this study to investigate COVID-19 contagion concern and perceived changes of family and peer relationships and of learning skills.

#### COVID-19 Contagion Concern

*COVID-19 contagion concern* was investigated through three questions asking “How worried are you that you/your relatives/your friends are getting sick because of the COVID-19?”, each one on a 4-point Likert scale (0 = not at all −3 = very much). A composite score of *COVID-19 contagion concern* was calculating by summing the three items (range 0–9) (Cronbach's alpha = 0.81).

#### Perception of Change of Family and Peer Relationships and of Learning Skills

Students were asked if their family relationships, peer relationships, and learning skills changed during lockdown. Each item was evaluated on a 10-points scale (from 0 = worsened a lot to 10 = improved a lot; midpoint of the scale 5 = no change). For descriptive analysis, each answer was codified in 3 categories, namely worsening (score 0–4), no change (score 5), and improvement (score 0–6). For the regression analysis, each answer was considered as a continuous variable, it was reversely coded and defined as “worsening of family relationships,” “worsening of peer relationships,” and “worsening of learning skills.”

#### Depressive Symptoms

Students completed the Italian version of the CESD-10 (Pierfederici et al., 1982) which evaluates the frequency of depressive symptoms during the past week on a 4-point Likert scale (0 = rarely/never- 3 = most/all of the time). A cut-off score of 10 indicates the presence of significant depressive symptoms (range 0–30, Cronbach's alpha = 0.84).

#### Emotional Self-Efficacy

*Emotional self-efficacy* was evaluated through the Multidimensional Negative Regulatory Emotional Self-Efficacy Scale (Caprara et al., 2013) composed of 9 items which evaluate the perceived ability to regulate negative affect (anger, sadness, and fear) on a 5-point Likert scale (1 = not able at all- 5 = very able) (range 9–45, Cronbach's alpha = 0.85).

#### Academic Self-Efficacy

The scale of Bandura et al. (1996) evaluates the students' perceived ability of self-regulating learning activities, asking teachers and peers for help and motivating themselves to study. Some questions were slightly modified to be adapted to the specific characteristics of remote learning (11 items on a 5-point Likert from 1 = not able at all to 5 = very able) (range 11–55, Cronbach's alpha = 0.88).

### Data Analysis

The percentage of missing data was <5%. The MCAR (Missing Completely at Random) test (Little, 1988) was not statistically significant, indicating that missing data were completely at random. Therefore, the imputation was carried out with the EM (Expectation-Maximization) procedure. As for the first aim, descriptive analyses were carried out through frequencies, *t*-test for gender differences, Cohen's d as a measure of *t*-test effect size, and Pearson's bivariate correlations. As for the second aim, the four hypothesized mediation models were tested through the PROCESS SPSS-macro (Hayes, 2017). For each model, the statistical significance of the indirect effect was evaluated through a bootstrapping procedure (95% confidence intervals with 5,000 bootstrap samples). Confidence intervals that do not contain zero indicate a statistically significant indirect effect (mediation). The effect size was evaluated through the completely standardized indirect effect and interpreted as small (0.01), medium (0.09), and large (0.25) (Preacher and Hayes, 2008; Preacher and Kelley, 2011). Gender was included as covariate in all models to control for its effect on depressive symptoms. Statistical analyses were performed using SPSS 26.

## Results

### Descriptives

Participants were mainly concerned that COVID-19 may infect their relatives (to some extent and very much 74%), less their friends and themselves (to some extent and very much 48 and 28.4%, respectively) (Table 2).

**Table 2:** Frequencies distribution of COVID-19 contagion concern.

	**Not at all**		**A little**		**To some extent**		**Very much**	
	***N***	**%**	***N***	**%**	***N***	**%**	***N***	**%**
Concerned that COVID-19 may infect yourself	74	25	138	46.6	69	23.3	15	5.1
Concerned that COVID-19 may infect your relatives	13	4.4	64	21.6	127	42.9	92	31.1
Concerned that COVID-19 may infect your friends	31	10.5	123	41.6	98	33.0	44	14.9

As for perceived change of family and peer relationships and learning skills, the worsening was mainly related to learning skills, reported by almost half of the participants (47.3%), while in general peer relationships was perceived as stable (44.6%) and family relationships were perceived as improved (53%) or stable (31.8%) (Table 3).

**Table 3:** Frequencies distribution of perceived change in family and peer relationships and learning skills.

	**Improvement**		**No change**		**Worsening**	
	***N***	**%**	***N***	**%**	***N***	**%**
Family relationships	157	53	94	31.8	45	15.2
Peer relationships	94	31.8	132	44.6	70	23.6
Learning skills	48	16.2	108	36.5	140	47.3

Depressive symptoms mean score was 13 (*sd* = 5.6) and most participants (*N* = 200, 67.6%) reported a score beyond the critical cut-off (≥10). As for emotional and academic self-efficacy, mean scores were 24.3 (*sd* = 5.8) and 36.1 (*sd* = 7.4), respectively. Concerning gender differences, female students reported higher COVID-19 contagion concern, higher worsening of learning skills, higher depressive symptoms, and lower emotional and academic self-efficacy than male (Table 4).

**Table 4:** Descriptive statistics of study variables: means in the total sample and by gender.

	**Total** ***N* = 296**	**Female** ***N* = 246**	**Male** ***N* = 50**	***t (df)***	***p***	**Cohen's *d***
	***M (sd)***	***M (sd)***	***M (sd)***			
COVID-19 contagion concern	4.6 (2.2)	4.9 (2.0)	3.1 (2.2)	5.9 (294)	0.0001	0.86
Worsening family	4.2 (1.9)	4.2 (1.8)	4.2 (2.0)	−0.1 (294)	0.956	0.00
Worsening peer	4.7 (1.7)	4.7 (1.7)	4.9 (1.7)	−0.7 (294)	0.466	0.12
Worsening learning	5.6 (1.7)	5.7 (1.7)	5.1 (1.9)	2.3 (294)	0.020	0.33
Depressive symptoms	13 (5.6)	13.6 (5.7)	9.9 (4.2)	4.4 (294)	0.0001	0.74
Emotional self-efficacy	24.3 (5.8)	23.3 (5.3)	29 (5.5)	−6.8 (294)	0.0001	1.05
Academic self-efficacy	36.1 (7.4)	35.7 (7.4)	38.3 (7.1)	−2.3 (294)	0.024	0.36

Results of correlation analysis showed that depressive symptoms were positively correlated with COVID-19 contagion concern, worsening of family relationships and worsening of learning skills and negatively correlated with both emotional and academic self-efficacy. Moreover, emotional self-efficacy was negatively correlated with COVID-19 contagion concern and worsening of learning skills. Academic self-efficacy was negatively correlated with worsening of learning skills (Table 5).

**Table 5:** Bivariate correlations between study variables.

		**1**	**2**	**3**	**4**	**5**	**6**	**7**
1	Depressive symptoms	–						
2	COVID-19 contagion	0.18^**^	–					
	concern							
3	Worsening family	0.24^**^	−0.04	–				
4	Worsening peer	0.11	−0.10	0.19^**^	–			
5	Worsening learning	0.32^**^	0.03	0.17^**^	0.12^*^	–		
6	Emotional self-efficacy	−0.55^**^	−0.32^**^	−0.10	−0.02	−0.15^*^	–	
7	Academic self-efficacy	−0.42^**^	−0.08	−0.13	−0.03	−0.60^**^	0.36^**^	–

* *p < 0.05 and*

** *p < 0.01*.

Results of correlation analysis showed that worsening of peer relationships was not related either to depressive symptoms, or emotional self-efficacy, thus the relation between worsening of peer relationships and depressive symptoms mediated by emotional self-efficacy was not tested.

### Mediation Analysis

**Figure 1:**
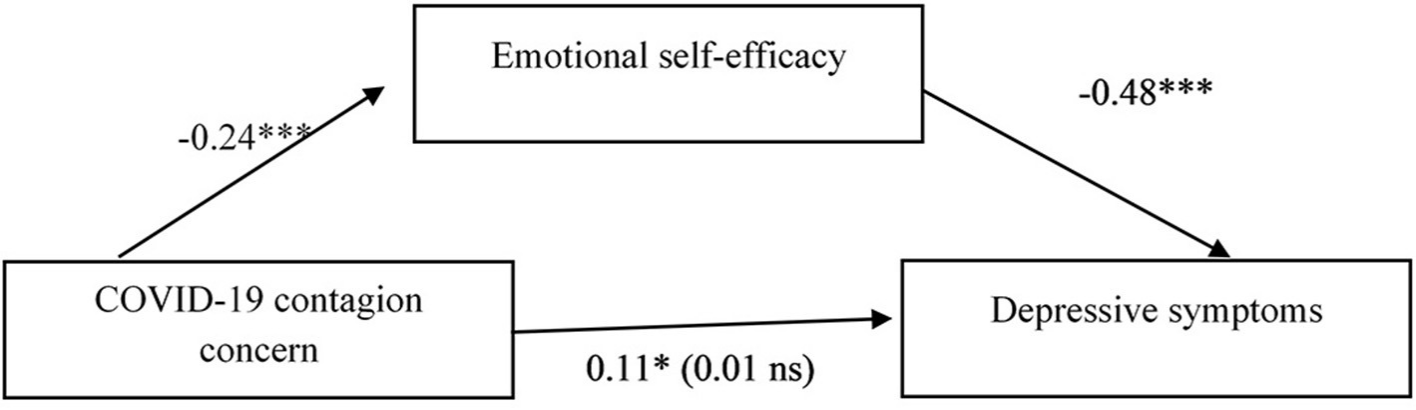
Standardized regression coefficients (beta) for the relation between COVID-19 contagion concern and depressive symptoms as mediated by emotional self-efficacy. ^*^p < 0.05; ^**^p < 0.01; and ^***^p < 0.001. In parenthesis the standardized regression coefficient between the independent variable and the dependent variable controlling for the mediator Gender beta = −0.21^***^. *R*^2^ = 0.26; *F*_(3, 292)_ = 34.57, *p* < 0.001.

The first model tested the relation between COVID-19 contagion concern and depressive symptoms mediated by emotional self-efficacy. Higher COVID-19 contagion concern was related to lower emotional self-efficacy and lower emotional self-efficacy to higher depressive symptoms. The indirect effect of COVID-19 contagion concern on depressive symptoms was statistically significant (*b* = 0.30, 95% CI [0.14, 0.48]) thus confirming the hypothesized mediating role of emotional self-efficacy. The completely standardized indirect effect indicated a medium effect size (Table 6 and Figure 1).

**Table 6:** Mediation effects in the tested models.

		**Bootstrapping 95% CI**	
		**Lower**	**Upper**
**Predictor: COVID-19 contagion concern**			
Direct effect	−0.01	−0.29	0.28
Indirect effect (emotional self-efficacy)	0.30	0.14	0.48
Completely standardized indirect effect (emotional self-efficacy)	0.11	0.06	0.18
**Predictor: worsening family**			
Direct effect	0.67	0.38	0.96
Indirect effect (emotional self-efficacy)	0.04	−0.10	0.20
Completely standardized indirect effect (emotional self-efficacy)	0.02	−0.03	0.06
**Predictor: worsening learning**			
Direct effect	0.44	0.06	0.82
Indirect effect (emotional self-efficacy)	0.13	−0.04	0.32
Indirect effect (academic self-efficacy)	0.39	0.15	0.65
Completely standardized indirect effect (emotional self-efficacy)	0.04	−0.01	0.10
Completely standardized indirect effect (academic self-efficacy)	0.12	0.05	0.20

**Figure 2:**
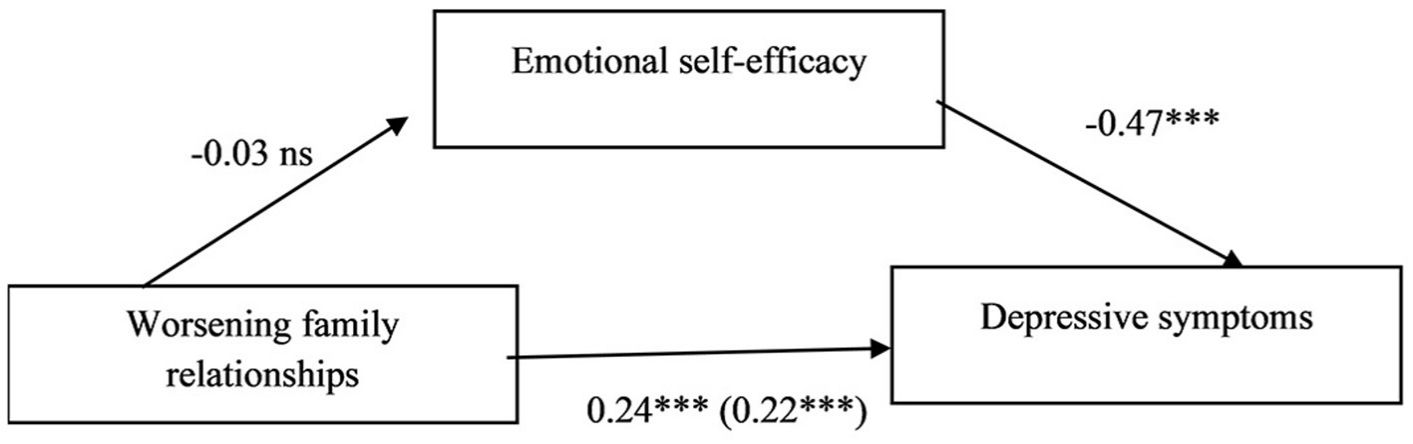
Standardized regression coefficients (beta) for the relation between worsening in family relationships and depressive symptoms as mediated by emotional self-efficacy. ^*^p < 0.05; ^**^p < 0.01; and ^***^p < 0.001. In parenthesis the standardized regression coefficient between the independent variable and the dependent variable controlling for the mediator Gender beta = −0.25^***^. *R*^2^ = 0.31; *F*_(3, 292)_ = 44.00, *p* < 0.001.

The second model tested the relation between worsening of family relationships and depressive symptoms mediated by emotional self-efficacy. Lower emotional self-efficacy was related to higher depressive symptoms, whereas worsening of family relationships was unrelated to emotional self-efficacy. The hypothesis of a mediation effect of emotional self-efficacy between worsening of family relationships and depressive symptoms was not confirmed, because the indirect effect was not statistically significant (*b* = 0.04, 95% CI [−0.10, 0.20]) (Table 6 and Figure 2).

**Figure 3:**
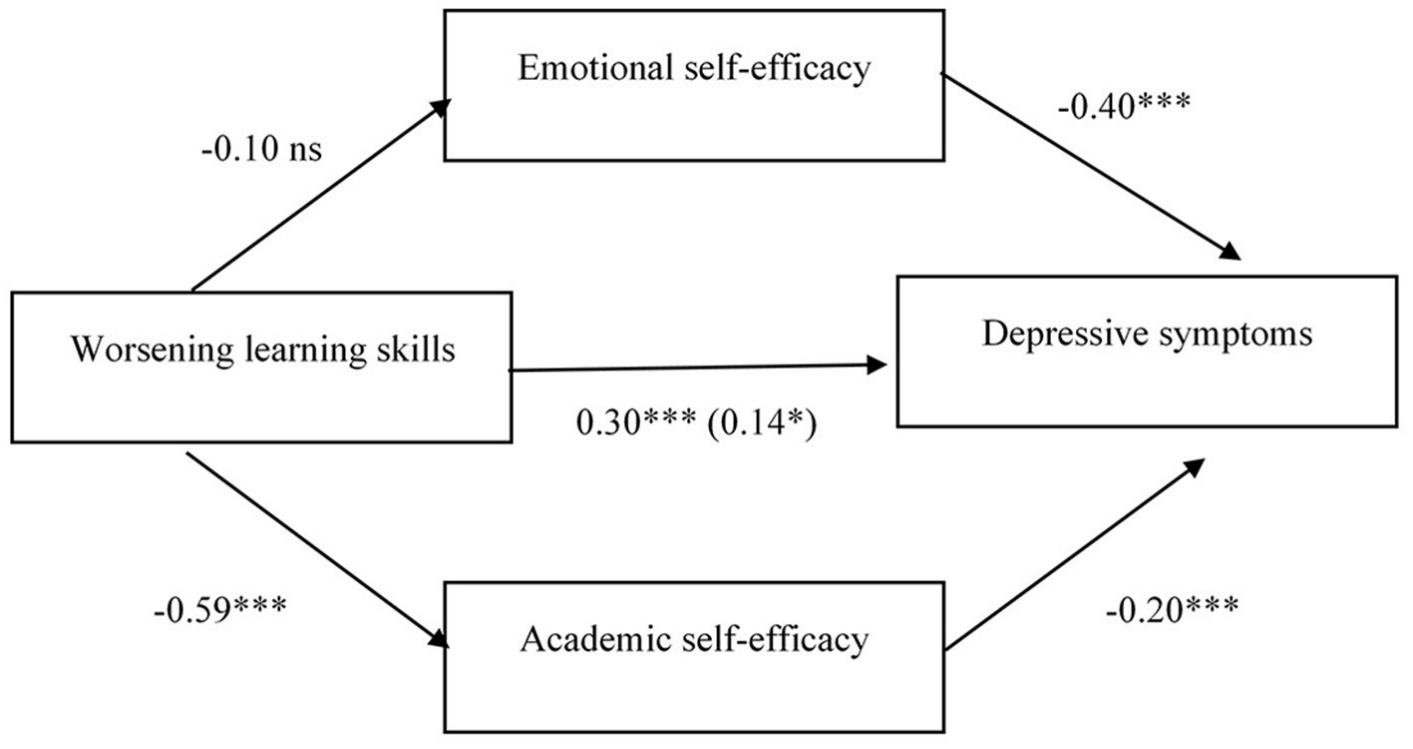
Standardized regression coefficients (beta) for the relation between worsening in learning skills and depressive symptoms as mediated by emotional and academic self-efficacy. ^*^p < 0.05; ^**^p < 0.01; and ^***^p < 0.001. In parenthesis the standardized regression coefficient between the independent variable and the dependent variable controlling for the mediator Gender beta = −0.21^***^. *R*^2^ = 0.35; *F*_(4, 291)_ = 38.65, *p* < 0.001.

Finally, the third model tested the relation between worsening of learning skills and depressive symptoms mediated by both emotional and academic self-efficacy. Worsening of learning skills was related to lower academic self-efficacy, whereas the relation with emotional self-efficacy was not statistically significant. Lower emotional and academic self-efficacy were both related to higher depressive symptoms. The indirect effect of worsening of learning skills on depressive symptoms was statistically significant through academic self-efficacy (*b* = 0.39, 95% CI [0.15, 0.65]), but not through emotional self-efficacy (*b* = 0.13, 95% CI [−0.04, 0.32]). Therefore, the hypothesized mediating role was confirmed only for academic self-efficacy. The completely standardized indirect effect indicated a medium effect size (Table 6 and Figure 3).

## Discussion

The present study explored how COVID-19 lockdown affected academic activities and learning skills, as well as relationships with family and peers and mental health in a group of Italian university students. The study timely investigated some of the consequences of the lockdown during the first phase of the COVID-19 pandemic (from March to May 2020) focusing on an age group still neglected in literature.

As for the first aim of our study, the descriptive results indicated a high prevalence of depressive symptoms, in line with other studies on the consequences of the COVID-19 pandemic and lockdown on young people's mental health (Liang et al., 2020; Meda et al., 2021). The high prevalence of depressive symptoms observed in our study (67% of the participants obtained a score beyond the CESD critical cut-off) deserves some considerations. It is worth noting that the reported prevalence of depressive symptoms largely varies in different studies on depression during COVID-19 pandemic, in relation to the psychological measures employed, as well as to demographic characteristics of participants. In particular, we found percentages varying from around 40% (Zhou et al., 2020) to 50% (Lan et al., 2020) up to around 60% (Solomou and Constantinidou, 2020).

Pre-pandemic studies generally reported a prevalence of depressive symptoms in the university population of around 30% (Liu et al., 2019; de Paula et al., 2020) and this percentage was also found among university students in Italy (Bert et al., 2020; Boietti et al., 2020). Therefore, the high rate of depressive symptoms found in our study seems to be in line with evidence that during the first phase of COVID-19 pandemic depression rate has increased significantly compared to the pre-pandemic period (Meda et al., 2021) and in some cases this prevalence rate has doubled or even tripled (Ettman et al., 2020).

Moreover, our result should not be considered so surprising, when considering some contextual factors of our study. First, data were gathered in May 2020, after a long and stringent period of lockdown (more than 2 months) that involved all the national territory and duration of isolation has been found to be correlated with mental health symptoms (Loades et al., 2020) also in previous pandemics (Brooks et al., 2020). Most of the respondents are resident in northern Italy, the part of the country hardest hit during the first phase of the pandemic, in terms of infection and mortality rates (Istituto Superiore di Sanità, 2020). Moreover, in Italy the lockdown was prolonged several times, with successive government decisions, resulting in great uncertainty. As outlined in the study of del Valle et al. (2020), greater uncertainty has proven to be related to higher anxiety and depression, especially among young women.

A final remark concerns the specific situation of Italian students. Universities in northern Italy had already been closed by the end of February 2020 and were not reopened before the end of the academic year. Students did not know for a long time whether they would attend the face-to-face classes again before the end of the year and what the situation would be for the new academic year. This great uncertainty was linked both to the particular moment and, more generally, to how studies would continue in the future. As mentioned before, uncertainty is supposed to have largely increased students' concerns and depressive symptoms.

The higher prevalence of depression among female students is an expected result, in line with studies reporting a higher risk for women to develop depression, both in normative and non-normative situations (Beirão et al., 2020; Ettman et al., 2020), thus gender differences seem to be maintained during the pandemic.

As for the other variables examined in our study, the descriptive analysis revealed that concerns related to COVID-19 contagion were widespread. This result was expected, especially due to the high spread of the virus in Italy during the period in which data were collected. The students were more concerned about contagion for their relatives than for themselves. This result can be linked to evidence that young age is a protective factor for COVID-19 complications, whereas the disease more seriously affects people over 60 (Istituto Superiore di Sanità, 2020). Moreover, young adults experienced themselves as potentially dangerous to their loved ones and this is likely to have increased their concerns for relatives. Worries about one's own health and the health of relatives were identified as main pandemic stressors among college students also in other studies (Son et al., 2020; Yang et al., 2021).

The disruption of daily routines caused by lockdown seems to have had an effect especially on academic activities, more than on peer and family relationships. Almost half of the students perceived a worsening of their learning skills during lockdown, and this is a noteworthy result, highlighting the serious effects of remote learning on students' perception of their abilities. Many factors were likely to contribute to the perception of this deterioration in study activities: the lack of appropriate devices or Internet connection lines, the absence of face-to-face relationships with teachers and classmates, the reduced motivation in following lessons, as well as the difficulties linked to the new examination procedures (Biwer et al., 2021; Nguyen et al., 2021).

On the contrary, relationships with peers and family seem to have been generally preserved from the negative effects of lockdown. In fact, most students reported stable peer relationships and stable or even improved family relationships. On the one hand, young people are used to keeping in touch with their friends virtually and therefore this dimension of life is likely to have been experienced as a less radical change and has generated fewer problems (Hamilton et al., 2020), especially if compared to academic activities. On the other hand, our interpretative suggestion is that during lockdown young people were likely to give less importance to friendships and focused on those aspects that could be preserved in this period: study and family relationships.

A possible reason to explain our results about the overall good quality of family relationships during lockdown can be found in the fact that the family has a pivotal role in the Italian context in terms of affective bonds, practical, and emotional support and most Italian young adults live with parents (Bonino and Cattelino, 2012). It is likely that for some participants the prolonged cohabitation with relatives was an opportunity to strengthen family ties and to experience reciprocal support and this was in turn related to better mental health as found in other studies (Ellis et al., 2020). In contrast, for a minority of participants, the forced cohabitation with their parents throughout the day and for such a long period of time may have caused conflict, stress and a perception of reduced autonomy (Fioretti et al., 2020), resulting in the experience of a deterioration of family relationships. Reduced autonomy collides with one of the main developmental tasks of young adults, namely the need for independence (Arnett, 2000). Italian university students, although often still living with their parents, realize their autonomy through extra-domestic activities: engagement in university, leisure activities, friendship, and couple relationships; all these aspects have been suddenly interrupted and prohibited by lockdown measures.

As for the second aim of our study, our hypotheses were partially confirmed. As hypothesized, COVID-19 contagion concerns were positively related to depressive symptoms, in line with the study of Montano and Acebes (2020), stressing the high vulnerability of young people to COVID-19 fear and to its implications on depression, anxiety, and stress symptoms. As expected, the association between COVID-19 contagion concern and depressive symptoms was mediated by emotional self-efficacy, suggesting that students who perceived themselves as more able to manage negative emotions were more likely to limit concern about COVID-19 contagion and to reduce its effect on depressive symptoms. During this ongoing pandemic, the burden of negative emotions is particularly heavy, therefore it is crucial to feel confident in one's abilities to manage them.

Regarding family relationships, as hypothesized students perceiving a worsening of family relationships reported higher depressive symptoms, but we did not find a mediating role of emotional self-efficacy. As previously said, only a minority of students experienced a worsening of family relationships. It is plausible that for these students the forced cohabitation in the family and the prohibition of experiences outside the home led to a worsening of family relationships and in turn to an increase in depressive feelings, irrespective of the presence of individual abilities, such as the confidence in managing negative emotions. However, our data do not allow to know the reasons of students' perception of a worsening of family relationships and further research investigating this aspect would be needed. Contrary to our expectations, a worsening of peer relationships was not associated with depressive symptoms. As previously stated, during lockdown friendships were likely to be maintained online and social media might have helped young people to cope with loneliness and psychological difficulties, as suggested in other studies (Cauberghe et al., 2021). Aspects concerning friendships and adaptation to lockdown among university students would also require further investigation.

Finally, as hypothesized, we found a higher risk of depressive symptoms for students reporting a worsening of learning skills. This finding suggests the extent to which the disruption of learning habits has been related to depressive feelings, as also outlined in other studies (Besser et al., 2020; Yang et al., 2020). As expected, the association between worsening of learning skills and depressive symptoms was mediated by academic self-efficacy: in other words, university students perceiving themselves as more able to manage their learning activities during home confinement were more likely to experience lower depressive symptoms. For these students, the ability of adapting to a new learning environment and new academic demands has been a resource for adaptively addressing the challenge of remote learning during lockdown.

The study has some limitations. First, participants were recruited using a convenience sampling approach and female, psychology students and first-year students were over-represented, thus limiting the generalizability of our results. Second, a selection bias may be linked to the use of online surveys. In particular, students who have less access to electronic devices and therefore experienced more difficulties during the lockdown are more likely to have been excluded from the study. Nonetheless, the use of snowball sampling through the Internet and the prevalence of female respondents are two features common to most studies published to date on the psychological effects of COVID-19 and related containment measures. Even though our participants were not representative of the population under study, the present research makes an important initial contribution to the knowledge that the scientific community is building about the psychological effects of this ongoing pandemic. Third, analysis with cross-sectional data could not establish causality and a more complex model of analysis could be used to analyze mediation in future studies. In fact, it is plausible that also depressive symptoms might have contributed to students' concern and perception of a worsening of their learning skills.

Despite these limitations, this study extends our understanding of the impact of the COVID-19 confinement on mental health of university students, an age group still under investigated, particularly in the Italian context. A further strength lies in the fact that our study has considered the role of family which is generally more studied with respect to childhood and less investigated in the case of young adults. This aspect is worth exploring in the Italian context, where family relationships are particularly significant not only for children and adolescents but also for young adults (Inguglia et al., 2015; Smorti et al., 2020). The high prevalence of depressive symptoms found in our study suggests that high attention should be paid to the psychological consequences of the pandemic for university students, especially to timely identify students more at risk for depressive symptoms. This knowledge is useful to develop and disseminate appropriate preventive interventions for a specific population largely neglected by Italian policies and media during the evolution of the pandemic. More broadly, the impact of the pandemic on university students in terms of mental health should be considered in the political choices for pandemic management.

The protective role of emotional and academic self-efficacy should be considered as an aspect to be enhanced to make students better equipped to face challenges. Our findings suggest that it is necessary for teachers to implement remote learning while maintaining stimulating activities and promoting social contacts, even if virtual, to support students' motivation and promote their self-efficacy beliefs. These objectives are difficult to achieve in an emergency condition, but they must be considered in planning future academic activities in the post-COVID-19 period (Nguyen et al., 2021; Rabaglietti et al., 2021). In particular, in view of a gradual resumption of a new “normality,” it will be important that remote learning and teaching processes will be planned according to the recommendations on online learning indicated in literature (Kearns, 2012). We believe that the results of our study should be considered in the post-COVID-19 recovery phase to promote the empowerment of university students who represent a population group particularly affected by the psychological effects of the pandemic and lockdown.

## Data Availability Statement

The raw data supporting the conclusions of this article will be made available by the authors, without undue reservation.

## Ethics Statement

The studies involving human participants were reviewed and approved by Comitato di Bioetica dell'Università degli Studi di Torino. The patients/participants provided their written informed consent to participate in this study.

## Author Contributions

ECal conceived the study, provided statistical analysis and interpretation of the data, and wrote the manuscript. FG participated in the study design and coordination, provided statistical analysis and interpretation of the data, and wrote the manuscript. TB participated in the study design, contributed to data collection, and collaborated in writing introduction of the manuscript and editing of the manuscript. ECat participated in the study design, contributed to data collection, and contributed to the interpretation of the results. SG participated in the study design, contributed to data collection, and collaborated to statistical analysis and interpretation. CR participated in the study design and contributed to data collection. AF participated in the study design, contributed to data collection, and to statistical analysis and interpretation. All authors read and approved the final manuscript.

## Conflict of Interest

The authors declare that the research was conducted in the absence of any commercial or financial relationships that could be construed as a potential conflict of interest.

## Publisher's Note

All claims expressed in this article are solely those of the authors and do not necessarily represent those of their affiliated organizations, or those of the publisher, the editors and the reviewers. Any product that may be evaluated in this article, or claim that may be made by its manufacturer, is not guaranteed or endorsed by the publisher.
